# Thermodynamic and transport properties of hydrogen containing streams

**DOI:** 10.1038/s41597-020-0568-6

**Published:** 2020-07-09

**Authors:** Aliakbar Hassanpouryouzband, Edris Joonaki, Katriona Edlmann, Niklas Heinemann, Jinhai Yang

**Affiliations:** 1 School of Geosciences, University of Edinburgh, Grant Institute, West Main Road, Edinburgh, EH9 3JW UK; 2 TÜV SÜD National Engineering Laboratory, Scottish Enterprise Technology Park, East Kilbride, South Lanarkshire G75 0QF United Kingdom; 3 Hydrates, Flow Assurance & Phase Equilibria Research Group, Institute of GeoEnergy Engineering, School of Energy, Geoscience, Infrastructure and Society, Heriot-Watt University, Riccarton, Edinburgh EH14 4AS UK

**Keywords:** Hydrogen energy, Underground hydrogen storage

## Abstract

The use of hydrogen (H_2_) as a substitute for fossil fuel, which accounts for the majority of the world’s energy, is environmentally the most benign option for the reduction of CO_2_ emissions. This will require gigawatt-scale storage systems and as such, H_2_ storage in porous rocks in the subsurface will be required. Accurate estimation of the thermodynamic and transport properties of H_2_ mixed with other gases found within the storage system is therefore essential for the efficient design for the processes involved in this system chain. In this study, we used the established and regarded GERG-2008 Equation of State (EoS) and SuperTRAPP model to predict the thermo-physical properties of H_2_ mixed with CH_4_, N_2_, CO_2_, and a typical natural gas from the North-Sea. The data covers a wide range of mole fraction of H_2_ (10–90 Mole%), pressures (0.01–100 MPa), and temperatures (200–500 K) with high accuracy and precision. Moreover, to increase ease of access to the data, a user-friendly software (H2Themobank) is developed and made publicly available.

## Abstract (summary)

**Table:** 

Measurement(s)	thermodynamic property • transport property
Technology Type(s)	computational modeling technique
Factor Type(s)	mole fraction of H2 • pressure • temperature

Machine-accessible metadata file describing the reported data: 10.6084/m9.figshare.12554804

## Background & Summary

To meet the Paris Agreement climate targets, global carbon emissions need to reach net-zero by 2050^1^. To achieve this the emissions from fossil fuels must be reduced and the energy mix transition to low carbon energy sources must be accelerated. Hydrogen can support this transition by replacing natural gas for domestic and industrial uses; replacing coal and natural gas for power generation; replacing fuel oil and gasoline to decarbonise transport and facilitating increased renewable energy by acting as an energy carrier to balance supply and demand. To enable hydrogen as a low carbon energy pathway, gigawatt-scale storage will be required^2–5^. Geological gas storage in underground salt caverns, depleted oil and gas fields and deep aquifers are proven technologies that could provide the necessary scales for hydrogen storage^6–8^. Hydrogen-rich town gas mixtures have been stored in geological formations since the 1970’s^9^ and currently, over 1,000,000 m^3^ of hydrogen is stored in underground salt caverns^10,11^. Furthermore, several types of gas have been successfully stored in geological formations, such as natural gas, compressed air and CO_2_. Recent work has shown that leakage of injected and stored gas is unlikely, if rigorous standards are in place^12^.

The storage of gas in the subsurface as chemical energy storage, whether as natural gas or hydrogen (the working gas), requires a cushion gas (30−70% of the total gas storage volume^13^) to prevent brine from entering the production stream and to maintain the required reservoir pressure ensuring deliverability. As depleted gas fields are being considered as storage sites for subsurface hydrogen storage, the *in situ* gas could be used as cushion gas and hence the working and cushion gasses will be of different compositions^7^. For gas storage in saline aquifers, where there is very little *in situ* gas present, there is a requirement to use a cushion gas that is significantly cheaper than the working gas. Considered options for aquifer storage cushion gasses are nitrogen, due to its low price, and CO_2_ due to its high compressibility and potential for secure storage of this greenhouse gas^14–16^. During the injection/production cycles, mixing of the gas components is inevitable and is determined by parameters such as mobility ratios, density differences, molecular diffusion and mechanical dispersion^17^. The numerical simulation of any storage scenario must confirm that the working gas can be produced with minimal cushion gas contamination. Therefore, if the cushion gas and working gas are of different compositions, the accurate quantification of the cushion gas/working gas mixing zone is of paramount importance. Once mixing takes place, the different gaseous components will alter the properties of the gas and introduce significant uncertainty into the expected behaviour of the injected, stored and produced gas, as shown for different gas storage applications^18,19^.

**Fig. 1:**
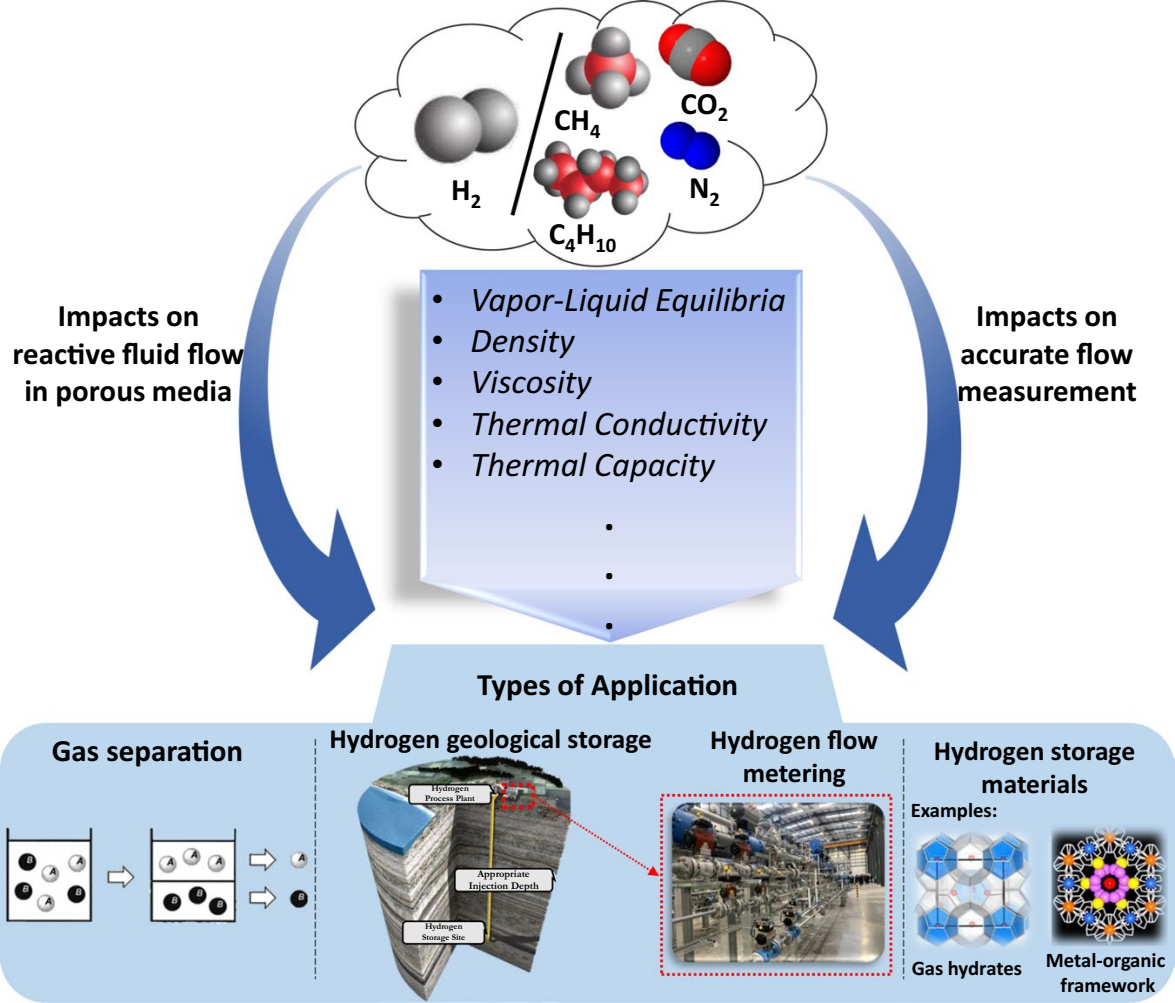
Potential applications of thermodynamic properties of hydrogen-containing streams for geological hydrogen storage, flow metering, gas separation^62^ and other types of hydrogen storage purposes such as gas hydrates^63,64^ or metal-organic framework^65^.

For gas storage modelling, accurate thermodynamic reference data for relevant fluid mixtures, which can either be directly imported into fluid flow modelling software or can be used to confirm existing reservoir engineering software outputs, is an important tool to enhance the compliance for scenario modelling results. Furthermore, the thermodynamic data for hydrogen-containing systems can enable scientists to have a deeper understanding of reactive flow through porous media during the hydrogen storage process. Another target in a hydrogen-based economy is to establish a fundamental understanding of metering technologies and the flow measurement principles behind them. In this regard, the thermo-physical properties of hydrogen mixed gases are crucial to understand and model hydrogen transportation and flow measurement processes. Thermo-physical properties of hydrogen-containing gas mixtures over a wide range of pressures and temperatures are pivotal to the design and optimisation of hydrogen production units, transportation, and storage processes (see Fig. 1).

Significant effort has been made to investigate the thermodynamic properties of hydrogen-containing mixtures systematically^20–35^. In addition, the phase equilibria and solubility of Hydrogen-natural gas components contained blends have been studied by researchers (see Table 1).

**Table 1:** Database of available experimental thermodynamic properties data in the literature for hydrogen-containing systems along with the temperature, pressure and composition range with respect to hydrogen for each binary/ternary system.

No.	System: H_2_ (1^st^ Component) + X (2^nd^ Component and beyond)	Property Type	Pressure Range (MPa)	Temperature Range (K)	x_1_ range (1^st^ component liquid mole fraction)	y_1_ range (1^st^ component gasmole fraction)	Reference
1	CH_4_	VLE/Solubility/Density/Viscosity/Compressibility/Thermal Conductivity	0.22–141.40	66.89–350.00	0.002–0.859	0.034–1.000	^20–35^
2	C_2_H_6_	VLE/Compressibility	0.27–562.50	83.00–283.15	0.002–0.800	0.085–1.000	^26,32,71–75^
3	C_3_H_8_	VLE/Compressibility	0.69–55.16	93.15–366.40	0.001–0.669	0.110–0.999	^26,71,76–79^
4	C_4_H_10_	VLE/Solubility	2.07–53.43	144.26–394.25	0.008–0.341	0.213–0.999	^20,80–82^
5	C_5_H_12_	VLE/Solubility	0.69–27.59	273.15–463.15	0.004–0.259	0.373–0.997	^83,84^
6	C_6_H_14_	VLE/Solubility	1.24–68.95	277.59–506.48	0.011–0.700	0.100–0.998	^85–87^
7	Cyclo-C_6_H_14_	VLE/Solubility	0.10–69.04	293.15–523.15	0.000–0.367	0.549–0.997	^83,88–96^
8	N_2_	VLE/Solubility/Heat Capacity/Compressibility	0.13–101.33	20.10–122.04	0.012–0.620	0.082–1.00	^20,28,31,34,97–113^
9	CO_2_	VLE/Viscosity/Density/Thermal Conductivity	0.93–191.80	219.90–298.15	0.001–0.744	0.043–0.934	^108,114–118^
10	H_2_S	Solubility	1.01–5.07	243.15–273.15	0.002–0.020	0.322–0.910	^119,120^
11	CO	VLE/Viscosity/Thermal Conductivity/Density	0.13–5.07	20.10–122.04	0.012–0.731	0.082–1.00	^27,110,121,122^
12	CH_4_ + C_2_H_6_	VLE	0.27–562.50	83.00–283.15	0.002–0.800	0.085–1.000	^72^
13	C_3_H_8_ + CO	VLE	0.69–20.68	88.15–348.15	0.005–0.107	0.034–0.847	^79^
14	CH_4_ + CO_2_	VLE	6.90–27.60	227.35–258.15	0.004–0.259	0.373–0.997	^84^
15	CH_4_ + CO	VLE	2.90–5.00	120.00–140.00	0.000–0.110	0.000–0.926	^31^
16	C_5_H_12_ + CO_2_	VLE	6.90–27.60	273.15–323.15	0.004–0.259	0.373–0.997	^84^
17	N_2_ + CO	VLE	0.003–22.80	58.15–122.04	0.012–0.930	0.082–1.00	^100,109–111^
18	CH_4_ + N_2_	VLE	3.40–10.00	80.00–144.00	0.009–0.720	0.060–1.00	^98^

**Fig. 2:**
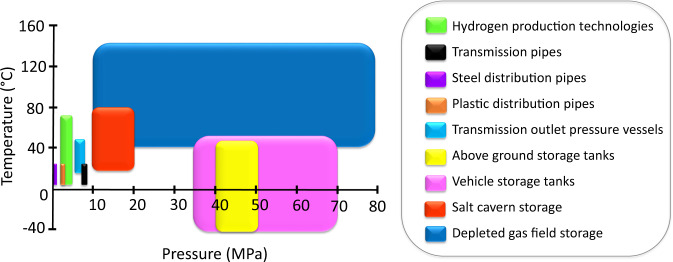
Pressure and temperature ranges for various hydrogen-based economy systems. Note that the pressure range for depleted gas fields is based on the data available for the UK only^66–69^.

While the thermodynamic properties of pure hydrogen are well established^36,37^, published properties of gas mixtures in relation to geological hydrogen storage^17,38–45^ do not cover the full range of additional gasses and often do not encompass the pressures and temperatures encountered within the hydrogen storage system (see Fig. 2). This data study will quantify the impacts of these additional gas components, to enable the accurate simulation of hydrogen mixed with various gases, all of which are essential for the modelling of the transportation, injection, geological storage, and production of hydrogen over multiple injection/production cycles.

## Methods

### Density and other derived thermodynamic properties using GERG-2008 Equation of State (EoS)

To efficiently design and operate the technical processes involved in gas-based energy industries, precise representation of the thermodynamic properties using an accurate EoS is essential. Here, the established and well regarded GERG-2008 EoS^46^ was used to predict phase behaviour and density of gas mixtures relevant to hydrogen storage, covering the thermodynamic properties of gas phase, liquid phase and supercritical regions. This equation is valid over a wide range of pressures and temperatures for 21 gas components including 1-methane, 2-nitrogen, 3-carbon dioxide, 4-ethane, 5-propane, 6-n-butane, 7-iso-butane, 8-n-pentane, 9-isopentane, 10-n-hexane, 11-n-heptane, 12-n-octane, 13-hydrogen, 14-oxygen, 15-carbon monoxide, 16-water, 17-helium, 18-argon, 19-n-nonane, 20-n-decane, and 21-hydrogen sulphide. The thermodynamic properties of the fluids that are predicted here at certain temperatures (*T*) are based on a multi-fluid approximation using the dimensionless Helmholtz energy obtained from:

1 $$\alpha (\delta ,\tau ,{\boldsymbol{x}})={\alpha }^{{\rm{o}}}(\rho ,T,{\boldsymbol{x}})+{\alpha }^{r}(\delta ,\tau ,{\boldsymbol{x}})$$

where *ρ* is the mixture density and ***x*** is the molar composition vector. The term $\tau =T/{T}_{r}$ is the inverse reduced temperature, and the term .. is the reduced density, both of which are composition-dependent i.e. they depend on the molar composition vector. The ideal-gas contribution (*α*^o^) is related to the number of mixture components (*N*), the mole fraction of each component i (${x}_{i}$), and the dimensionless Helmholtz energy of component i in the ideal-gas phase (${\alpha }_{{\rm{o}}i}^{{\rm{o}}}$) by:

2 $${\alpha }^{{\rm{o}}}(\rho ,T,{\boldsymbol{x}})=\mathop{\sum }\limits_{i=1}^{N}{x}_{i}[{\alpha }_{{\rm{o}}i}^{{\rm{o}}}(\rho ,T)+ln{x}_{i}]$$

The residual part of the dimensionless Helmholtz energy (*α*^r^) is composed of two different parts; the linear summation of the residual part of the reduced Helmholtz free energy of each component i (${\alpha }_{oi}^{r}$) and the so-called departure function (Δ*α*^*r*^) which is also a function of the mixture composition, the inverse reduced mixture temperature, and the reduced mixture density. The residual part of the dimensionless Helmholtz energy is given by:

3 $${\alpha }^{r}(\delta ,\tau ,{\boldsymbol{x}})=\mathop{\sum }\limits_{i=1}^{N}{x}_{i}{\alpha }_{oi}^{r}(\delta ,\tau )+\Delta {\alpha }^{r}(\delta ,\tau ,{\boldsymbol{x}})$$

The advantage of using Helmholtz energy in the given form is that all the other thermodynamic properties can be derived analytically from terms *α*° and *α*^*r*^ and their derivatives. One example is isobaric heat capacity which is given by:

4 $${c}_{p}(\delta ,\tau ,x)={\rm{R}}\left[-{\tau }^{2}({\alpha }_{\tau \tau }^{{\rm{o}}}+{\alpha }_{\tau \tau }^{{\rm{r}}})+\frac{{\left(1+\delta {\alpha }_{\delta }^{r}+\delta \tau {\alpha }_{\delta \tau }^{r}\right)}^{2}}{1+2\,\delta {\alpha }_{\delta }^{r}+{\delta }^{2}{\alpha }_{\delta \delta }^{r}}\right]$$

where R is the gas constant. The subscriptions of *α*° and *α*^*r*^ denote the order of their derivatives with respect to $\tau$ and $\delta$. For example ${\alpha }_{\tau \tau }^{\tau }$ denotes the second-order derivatives of *α*^*r*^ with respect to $\tau$. Similarly, enthalpy (h), entropy (s), Gibbs free energy (g), pressure (P) can be obtained from:

5 $$P(\delta ,\tau ,x)=RT\rho [1+\delta {\alpha }_{\delta }^{r}]$$

6 $${\rm{h}}\left(\delta ,\tau ,{\boldsymbol{x}}\right)={\rm{RT}}\left[1+\tau \,\left({\alpha }_{\tau }^{o}+{\alpha }_{\tau }^{r}\right)+\delta {\alpha }_{\delta }^{r}\right]$$

7 $${\rm{s}}\left(\delta ,\tau ,{\boldsymbol{x}}\right)={\rm{R}}\left[\tau \,\left({\alpha }_{\tau }^{{\rm{o}}}+{\alpha }_{\tau }^{r}\right)-{\alpha }^{{\rm{o}}}-{\alpha }^{{\rm{r}}}\right]$$

8 $${\rm{g}}\left(\delta ,\tau ,{\boldsymbol{x}}\right)={\rm{RT}}\left[1+{\alpha }_{\tau }^{{\rm{o}}}+{\alpha }_{\tau }^{r}+\delta {\alpha }_{\delta }^{r}\right]$$

Other thermodynamic properties such as compression factor, internal energy, speed of sound, Joule-Thomson coefficient, etc. can be defined similarly. Kunz. *et al*.^46^ provides comprehensive coverage of these derivatives and thermodynamic properties.In GERG-2008 EoS, terms ${\rho }_{r}$ and ${T}_{r}$ are calculated using quadratic mixing rules proposed by Klimeck^47^:

9 $$\frac{1}{{\rho }_{r}({\boldsymbol{x}})}=\mathop{\sum }\limits_{i=1}^{N}{x}_{i}^{2}\frac{1}{{\rho }_{c,i}}+\mathop{\sum }\limits_{i=1}^{N-1}\mathop{\sum }\limits_{j=i+1}^{N}\frac{2{x}_{i}{x}_{j}}{{\rho }_{c,ij}}$$

10 $${T}_{r}({\boldsymbol{x}})=\mathop{\sum }\limits_{i=1}^{N}{x}_{i}^{2}{T}_{c,i}+\mathop{\sum }\limits_{i=1}^{N-1}\mathop{\sum }\limits_{j=i+1}^{N}2{x}_{i}{x}_{j}{T}_{c,ij}$$

where ${\rho }_{c,i}$ is the critical density of component i, .. is the critical temperature of component i. The parameters for the components studied in this study are provided in figshare entry^48^. ${T}_{c,ij}$ and ${\rho }_{c,ij}$ are obtained from:

11 $$\frac{1}{{\rho }_{c,ij}}={\beta }_{\nu ,ij}{\gamma }_{\nu ,ij}\frac{{x}_{i}+{x}_{j}}{{\beta }_{\nu ,ij}^{2}{x}_{i}+{x}_{j}}\cdot \frac{1}{8}{\left(\frac{1}{{\rho }_{c,i}^{1/3}}+\frac{1}{{\rho }_{c,j}^{1/3}}\right)}^{3}$$

12 $${T}_{c,ij}={\beta }_{T,ij}{\gamma }_{T,ij}\cdot \frac{{x}_{i}+{x}_{j}}{{\beta }_{T,ij}^{2}{x}_{i}+{x}_{j}}{({T}_{c,i}{T}_{c,j})}^{1/2}$$

where *β*_*v*,*ij*_, *γ*_*v*,*ij*_, *β*_*T*,*ij*_, and *γ*_*T*,*ij*_ are the four adjustable binary interaction parameters. The binary interaction parameters used for the components in this study are provided in figshare entry^48^.

**Fig. 3:**
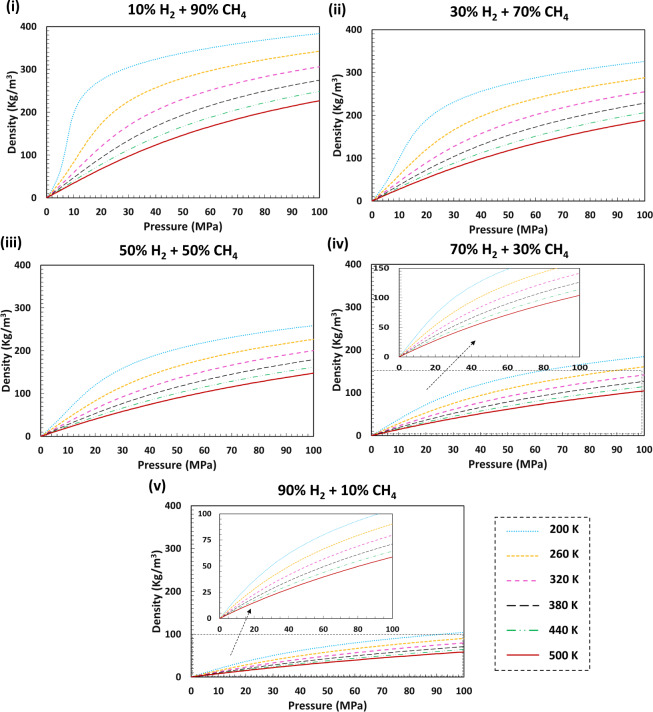
Predicted densities for different H_2_ + CH_4_ mixtures for various mole H_2_ fractions over a wide range of pressures and temperatures using GERG-2008 EoS. Density values are greater in the presence of higher mole fractions of CH_4_ in the studied systems as the density of CH_4_ is considerably higher than that of H_2_. The densities increase with increasing pressure (Boyle’s Law) for all isotherms and reduce with increasing temperature (Charles’s Law).

**Fig. 4:**
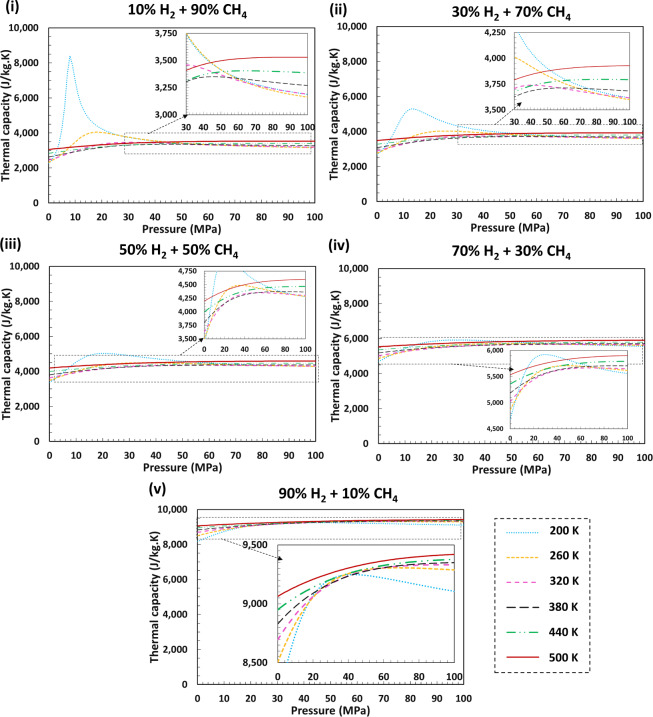
Predicted isobaric heat capacities for different H_2_ + CH_4_ mixtures for various H_2_ mole fractions over a wide range of pressures and temperatures using GERG-2008 EoS. Thermal capacities have higher values for higher H_2_ mole fractions as the heat capacity of pure H_2_ is significantly higher than that of pure CH_4_ at temperatures and pressures above the critical point of CH_4_. Generally, it can be noted that with increasing pressure, the thermal capacities increase for all temperature conditions due to increased intermolecular forces. The peaks in the graphs can be attributed to the fact that near the critical points of the components the heat capacities undergo sudden changes because of the changes in their phase. In these examples, as the temperatures and pressures are close to the critical conditions of CH_4_, peaks have emerged. Reducing the mole fraction of CH_4_ in the system composition moves the system away from the critical point and as such the peaks reduce or do not appear in the graphs (iv and v).

An example of the calculated densities and isobaric heat capacity for H_2_ + CH_4_ mixtures over a range of pressure and temperature for various H_2_ mole fractions is provided in Figs. 3 and 4, respectively. Plots of the other derived thermodynamic properties of H_2_ with CH_4_ and the thermodynamic properties of H_2_ with CO_2_, N_2_, and the typical natural gas are presented in figshare entry^48^.

### Viscosity and thermal conductivity using SuperTRAPP model

**Fig. 5:**
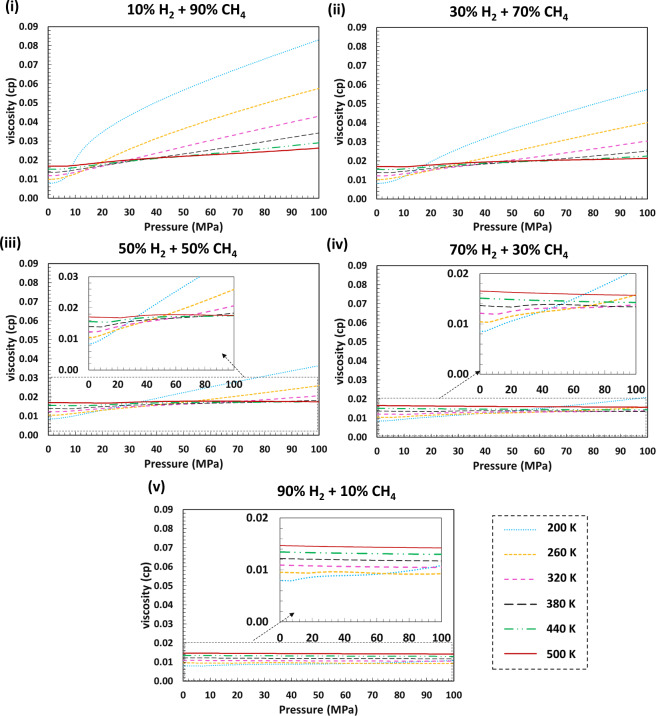
Modelled viscosity values for various H_2_ + CH_4_ blends for different H_2_ mole fractions over a wide range of pressures and temperatures using GERG-2008 EoS and SuperTRAPP model. The viscosities of the mixtures are suppressed with increasing H_2_ mole fractions in the system as H_2_ has a significantly lower viscosity than CH_4_ due to its smaller molecule size. The viscosities of the blends increase with increasing pressure and temperature. This can be attributed to the fact that an increase in pressure or temperature increases the velocities of the random motion of molecules and as such collisions of gas molecules increase, which resists the flow of gas and increases the viscosity. The unusual behaviour of CH_4_-rich blends at lower temperatures is because of their proximity to the CH_4_ critical point.

For calculating viscosity of the system we used SuperTRAPPmodel^49^ that is based on the corresponding-states model. SuperTRAPP viscosity model is composed of a dilute-gas and residual contribution part, where only the latter is treated with corresponding states. The viscosity (*μ*) is a function of density and pressure and is obtained from:

13 $$\mu (T,\rho )={{\rm{\mu }}}^{\ast }(T)+\Delta {\mu }_{0}\left({T}_{0},{\rho }_{0}\right){F}_{\mu }(T,\rho )$$

where * refers to dilute gas and 0 refers to a reference fluid. The dilute gas viscosity is calculated using Chung *et al*.^50^ theory which is a modification of the original model by Chapman-Enskog^51^. The function ${F}_{\mu }$ can be obtained from:

14 $${F}_{\mu }\left(T,\rho \right)=\sqrt{f}\,{h}^{-2/3}\sqrt{\frac{m}{{m}_{0}}}$$

where *m*, and *m*_0_ are the molar mass of the main fluid and the reference fluid, respectively. The terms *f* and *h* are so-called equivalent substance reducing ratios, relating the reference fluid to the studying fluid using critical parameter ratios. For a more detailed examination of the formulations used for calculating viscosity, the reader is referred to reference^49^. An example of calculated viscosities for H_2_ + CH_4_ mixtures over a range of pressures and temperatures for various H_2_ mole fractions is provided in Fig. 5. Plots of the viscosity of H_2_ with CO_2_, N_2_, and typical natural gas are presented in figshare entry^48^.To calculate the thermal conductivity of the fluids (*γ*) we also used SuperTRAPP model^49^. The thermal conductivity is obtained from:

15 $$\gamma \left(T,\rho \right)={{\rm{\gamma }}}^{int}\left(T\right)+\mathop{\overbrace{{{\rm{\gamma }}}^{\ast }\left(T\right)+{{\rm{\gamma }}}^{r}\left(T,\rho \right)+{{\rm{\gamma }}}^{crit}\left(T,\rho \right)}}\limits^{trans}$$

**Fig. 6:**
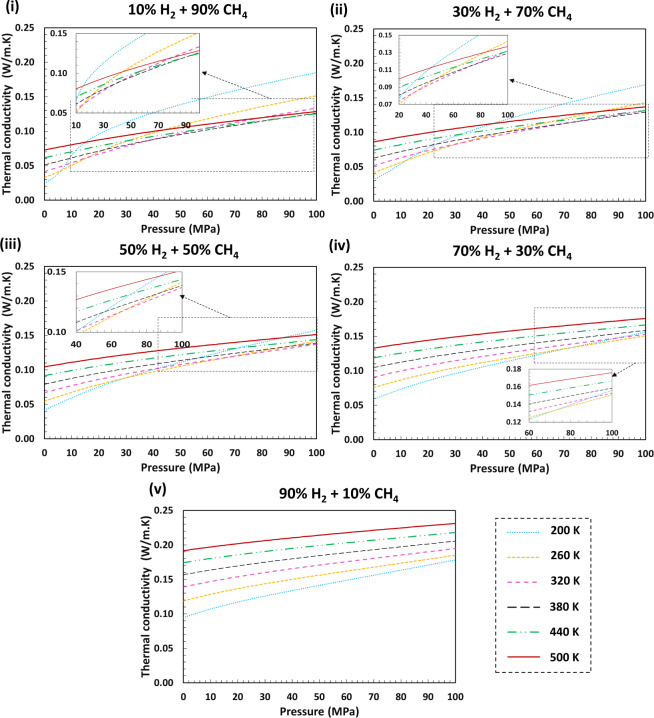
Estimated thermal conductivities for different H_2_ + CH_4_ blends with a range of H_2_ mole fractions over a wide range of pressures and temperatures using GERG-2008 EoS and SuperTRAPP model. It can be inferred that for all hydrogen/methane mixtures, the thermal conductivity values increase with increasing pressure for all isotherms. Thermal conductivities also increase with temperature for high H_2_ mole fraction systems (above 50%). These behaviours can be attributed to the fact that increasing pressure or temperature increases the molecular motion and as such improves the conduction of heat within gas molecules. The unusual behaviour of CH_4_-rich streams at lower temperatures is because of the proximity of these points to the CH_4_ critical point. Generally, thermal conductivity values increase with increasing hydrogen mole fractions as pure H_2_ has a considerably higher thermal conductivity than CH_4_.

This method is based on the Ely and Hanley procedure^52^ for calculating the thermal conductivity, where the model considers the effect of collisions between molecules (translational energy transfer) (γ^*trans*^), and the internal motions of the molecules (γ^*int*^, calculated using modified Eucken correlation). The former term can be further divided into three contributions i.e. dilute gas (γ^*^), residual (γ^*r*^) and critical enhancement (γ^*crit*^). We refer the reader to the article by Huber^49^ for detailed formulation and parameters of the thermal conductivity. An example of the calculated thermal conductivities for H_2_ + CH_4_ mixtures over a range of pressures and temperatures for various H_2_ mole fractions is provided in Fig. 6. The plots of thermal conductivity of H_2_ with CO_2_, N_2_, and the typical natural gas are presented in figshare entry^48^.

### Vapour liquid Equilibria and isothermal flash

**Fig. 7:**
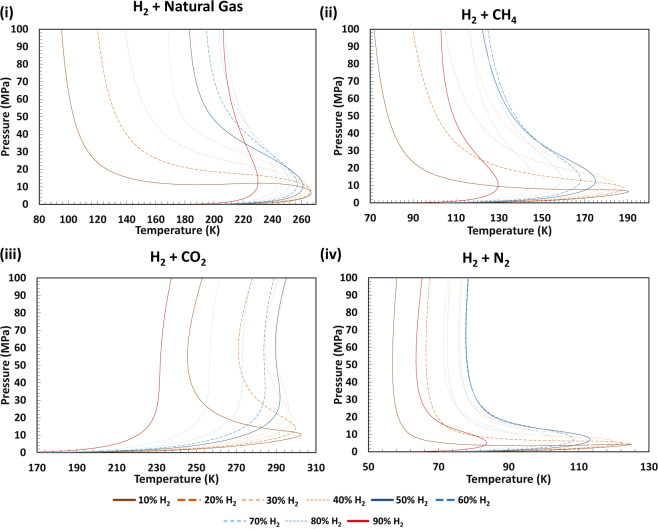
Modelled Vapour liquid Equilibria (VLE) diagrams using the developed tool in this study for various H_2_ containing mixtures with different H_2_ mole fractions over a wide range of pressures and temperatures. Comparing Fig. 2 with the above figure highlights that the possibility of entering into a two-phase region only exists in systems with higher CO_2_ concentrations. We refer readers to an excellent book on thermodynamics and phase behaviour of fluids to read more details about the behaviour of mixed fluids under various pressure and temperature conditions^70^.

For calculating phase equilibrium of the studied mixtures, we used a method established by Michelsen^53^. The vapour—liquid phase envelopes calculated for the system studied are provided in Fig. 7. As can be seen, some parts of two-phase envelope of the H_2_ + CO_2_ and the H_2_ + Natural gas systems occur within the pressure and temperature ranges of geological storage and as such are used in this study (temperature 200–500 K and pressure 0.01–100 MPa). For these points, we used isothermal multi-phase flash to calculate fraction and composition of gas and liquid phase. Here, we followed stability analysis by the successive substitution method which was introduced by Michelsen^54^ to minimise the Gibbs energy of the system. This was followed by the calculation of thermodynamic properties for each phase.

## Data Records

Viscosity, thermal conductivity, density, and other derived thermodynamic properties of H_2_ mixed with N_2_, CO_2_, CH_4_, and a typical natural gas from the UK North Sea (see Table 2) is provided for temperatures between 200–500 K, pressures between 0.01–100 MPa and mole fractions of hydrogen and additional gases between 10–90% using the described method. Note that the presence of water vapour, other impurities within the natural gas composition or selecting a different natural gas composition will affect the accuracy of the properties calculated.

**Table 2:** Natural gas molar composition used in this study.

Component	Mole%
CH_4_	83.60
C_2_H_4_	7.48
C_3_H_8_	3.92
n-C_4_	0.81
i-C_4_	0.81
n-C_5_	0.15
i-C_5_	0.14
N_2_	1.95
CO_2_	1.14

The composition is based on data obtained from a typical UK North Sea natural gas^123^.

The calculated data is publicly available and can be obtained using the following sources:

### H2ThermoBank

An open-source user-friendly software developed using C# code in visual studio to ease the access of data for any user.

### Excel format

The data is uploaded to (figshare) and is publicly accessible^48^.

### Figure format

Some selected data points with large steps are plotted and provided in figshare entry^48^.

## Technical Validation

The validity of the code developed for this study is checked by comparing the calculated results for a sample natural gas with existing data in the literature. Numerous pressure and temperature points were randomly selected for this comparison. The manual validation revealed no error in the written script for this study.

The GERG-2008 EoS is valid over a wide range of temperatures, pressures, and gas compositions achieving high accuracy in the prediction of thermodynamic properties of the 21 components listed in the methods section. Although the GERG-2008 EoS has been fitted to a wide range of experimental data, for some binary mixtures only the reducing functions were used. This is because predicting a general rule for accuracy of the GERG-2008 EoS for such binary mixtures is a very challenging task as there is no experimental data available for some ranges, therefore the absolute value of error for such ranges is considered to be unknown. However, the GERG-2008 EoS has been extensively compared with available data and its validity range can be divided into three pressure/temperature ranges:

- Normal range: which covers temperatures between 90 K and 450 K and pressures up to 35 MPa (The uncertainty of calculated density is less than 0.1% over the major part of this range)
- Extended range: which covers temperatures between 60 K and 700 K and pressures up to 70 MPa (The uncertainty of calculated density is less than 0.5% over the major part of this range)
- Extrapolated range: which covers temperatures and pressures beyond the previous range. (The uncertainty of calculated density is less than 1% over the major part of this range up to 100 MPa)

A number of studies using the same thermodynamic models have demonstrated the validity and accuracy of GERG-2008 EoS for different mixtures such as CH_4_ mixtures^55,56^, CO_2_ mixtures^57,58^, natural gas^59,60^ and compressed air^61^.

**Fig. 8:**
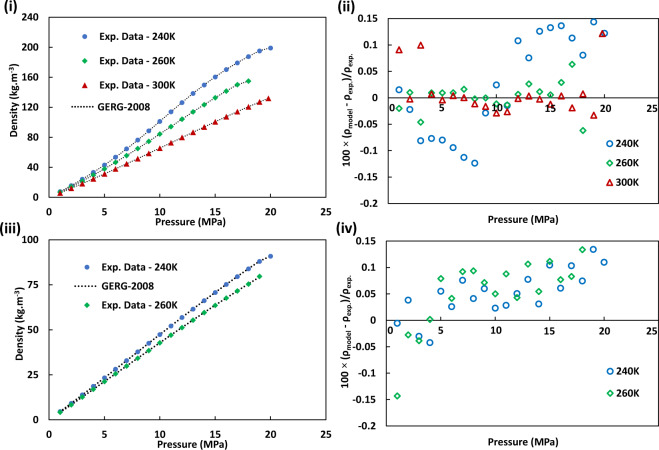
Thermodynamic modelling and experimental results of density of hydrogen/methane mixtures at a range of temperatures and pressures: i & ii are the results for a10%H_2_ + 90%CH_4_ mixture and iii & iv are the results for a 50%H_2_ + 50%CH_4_ mixture at different pressure and temperatures. ii and iv show the relative deviations in density values predicted by GERG-2008 equation of state, *ρ*_model_, from the density from the experimental (*ρ*_exp._) data^23^ versus pressure at different temperatures. The relative expanded uncertainties in experimental density (*k* = 2) *U*(*ρ*_exp_) for the10%H_2_ + 90%CH_4_ mixture and the 50%H_2_ + 50%CH_4_ mixture are 0.024 ≤ *U*(*ρ*_exp_) ≤ 0.046 and 0.024 ≤ *U*(*ρ*_exp_) ≤ 0.034, respectively.

We have utilised published density data of hydrogen/methane blends at a range of pressure and temperature conditions to statistically analyse and assess the reliability and accuracy of the attained modelling data from the GERG-2008 EoS. Figure 8 presents the relative deviations of the predicted densities of GERG-2008 EoS from the published hydrogen/methane blend experimental data^23^. The average absolute deviations (AADs) of the GERG-2008 EoS calculated data used in this study from the experimental data for the various pressures and temperatures are 0.044 for the 10%H_2_ + 90%CH_4_ mixture and 0.006 for the 50%H_2_ + 50%CH_4_ mixture. The low AAD values confirm the high accuracy of GERG-2008 EoS predictions with relatively low errors as discussed above.

The errors in viscosity and thermal conductivity estimates at various pressures, temperatures and gas compositions are uncertain due to the lack of experimental data. It is extremely time consuming and almost impossible to measure all the data required using existing laboratory methods. However, the thermal conductivity and viscosity of various compositions calculated from SuperTRAPP model have been compared with experimental data, with an error range of 0–15% reported in the literature^49^.

## Usage Notes

### H2ThermoBank

**Fig. 9:**
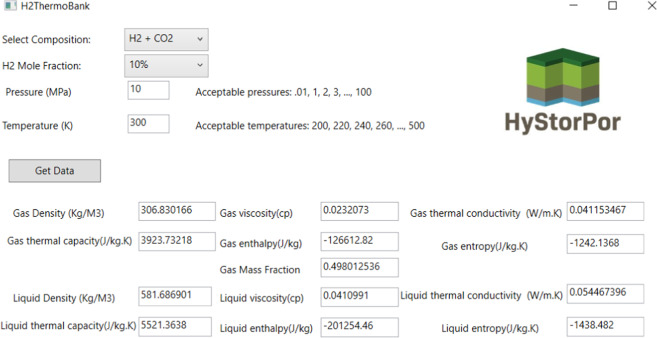
Example of graphical user interface of H2Thermobank. Here, we chose a mixture of CO_2_ and H_2_ with 10% H_2_ and 90% CO_2_, at 10 MPa and 300 K and clicked on the “Get Data” button. The image presents the calculated thermodynamic and transport properties of the liquid phase and the gas phase. In addition, the gas mass fraction for this mixture at the entered condition is obtained.

A screenshot of the data bank software is provided in Fig. 9. To calculate the required data, the user initially needs to select the gas composition of the closed system. There are 4 options (H_2_ + CH_4_, H_2_ + N_2_, H_2_ + CO_2_, and H_2_ + Natural gas). Following this, the H_2_ mole fraction in the closed system should be selected. After entering the desired pressure and temperature the “Get Data” button should be clicked to collect the data. For systems of H_2_ + CH_4_ and H_2_ + N_2_ there is only one phase exist in the covered range. It is important to note that for H_2_ + CO_2_ and H_2_ + Natural gas system some of the two-phase region is covered in this study (see Fig. 7). For these systems, the user will be able to get both liquid and gas properties together with the mass fraction of the gas phase.

In this study, we have utilised a newly developed tool based on the GERG-2008 EoS and SuperTRAPP model to predict different thermo-physical properties of hydrogen (e.g. density, viscosity, thermal conductivity, etc.) when mixed with other gaseous species including methane, nitrogen, carbon dioxide and a typical North Sea natural gas. The model has been applied to a wide range of pressures, temperatures, and gas mixture compositions which cover the temperature and pressure conditions experienced within the whole hydrogen-based energy system from production to storage in geological formations. The obtained results could be employed by a range of different stakeholders to effectually design and develop innovative infrastructure for the hydrogen economy.

### Excel format

To enable easy access to the data over a wide range of temperature, pressure and concentration conditions without requiring running the abovementioned application for each point, four excel files for each of gas mixture systems are provided. Each worksheet in the excel files is allocated to a different mole fraction of hydrogen. Data provided here could be sorted and selected for a required range.

## Data Availability

The code for H2ThermoBank has been made available on the H2ThermoBank GitHub page (https://github.com/aliakbarhssnpr/H2ThermoBank).
